# Analysis Method for Laterally Loaded Pile Groups Using an Advanced Modeling of Reinforced Concrete Sections

**DOI:** 10.3390/ma11020300

**Published:** 2018-02-15

**Authors:** Stefano Stacul, Nunziante Squeglia

**Affiliations:** Department of Civil and Industrial Engineering, University of Pisa, Largo Lucio Lazzarino, 56122 (PI) Pisa, Italy; squeglia@ing.unipi.it

**Keywords:** laterally loaded piles, pile group, shadowing effect, boundary element method, tension stiffening, reinforced concrete, suction

## Abstract

A Boundary Element Method (BEM) approach was developed for the analysis of pile groups. The proposed method includes: the non-linear behavior of the soil by a hyperbolic modulus reduction curve; the non-linear response of reinforced concrete pile sections, also taking into account the influence of tension stiffening; the influence of suction by increasing the stiffness of shallow portions of soil and modeled using the Modified Kovacs model; pile group shadowing effect, modeled using an approach similar to that proposed in the Strain Wedge Model for pile groups analyses. The proposed BEM method saves computational effort compared to more sophisticated codes such as VERSAT-P3D, PLAXIS 3D and FLAC-3D, and provides reliable results using input data from a standard site investigation. The reliability of this method was verified by comparing results from data from full scale and centrifuge tests on single piles and pile groups. A comparison is presented between measured and computed data on a laterally loaded fixed-head pile group composed by reinforced concrete bored piles. The results of the proposed method are shown to be in good agreement with those obtained in situ.

## 1. Introduction

The response to the horizontal loading of pile foundations, starting with the single pile, passing through the pile groups and finally to the combined piled-rafts has been the focus of many studies. However, as noted by many authors including Mokwa and Duncan [1] and Katzenbach and Turek [2], additional tests are needed to better understand the interactions between the soil, piles and superstructures. For the single pile case, it is well known that the key factors that influence the response include the restraint condition at the pile-head and the pile-soil relative stiffness.

For laterally loaded pile groups, full scale tests conducted by O’Neill [3] and Huang et al. [4] failed to provide definitive information about the influence of the execution technique while it is certain that the additional factors to be considered, compare to the single pile case, are: pile spacing, pile-soil-pile interactions, the stiffness of the connecting structure. 

Generally, it is assumed that in pile-groups, under horizontal loads, the displacement of the pile-heads is the same for all the piles and, so it is heterogeneous the load distribution between these. In general, because of group effects and in particular the shadowing effect [5] the efficiency of a pile-group is less than unity with a decreasing trend as the load increase. The efficiency achieves an asymptotic value for displacements larger than 0.06 pile diameter *D* [6]. Group effects such as the shadowing, which leads to a different load distribution between the piles in a group, tend to disappear for spacing values larger than 5–7 diameters [7,8].

The most common analysis methods are continuum-based or Winkler-based approaches (non-linear transfer curves or p-y curves). p-y curves methods are limited to the use of a subgrade soil reaction modulus which does not represent an actual property of the soil, and the soil is schematized with a series of independent springs that require the introduction of specific multipliers less than unity, [5], by which the transfer curves are scaled to consider the presence of group effects.

Since the introduction of the multipliers-concept a lot of works have been published [6,9,10] with the aim to determine a so+rt of project curves, for these coefficients, as a function of the spacing and the pile position in the group, although as evidenced by [11] would be more appropriate to evaluate the trend of these multipliers as a function of the load level (or the displacement level).

Some of the most common p-y curves (and implemented in software such as LPILE [12] GROUP [13], FLPIER [14]) include those recommended by the American Petroleum Institute. These were obtained from experimental tests on steel pipe piles with an outer diameter of about 30 cm, and which are not affected by the non-linearity of the pile material. Even recently new analytical methods based on the transfer curves have been developed [15,16] and new p-y curves were experimentally determined [17].

Continuum-based approaches are usually solved with boundary element methods (BEM) and finite element methods (FEM). Despite their potential in geotechnical engineering applications, FEM suffer from the complexity of the domain discretization, difficulties in choosing the input parameters and as evidenced in [18] FEM results are affected by the pile modeling. The high computational costs also prevent their use in parametric studies.

Often, therefore, they are used as a benchmark to validate other simplified approaches or as a tool to determine p-y curves for comparison with those obtained experimentally in situ [19,20].

BEM approaches, however, describe the soil as a homogeneous elastic half-space, characterized by a Young modulus and Poisson ratio, and enable pile-soil-pile interactions to be directly evaluated, and group effects can be considered.

These methods provide a complete solution at the interfaces of the problem domain but entail numerical approximations when the analysis involves heterogeneous soils. To evaluate the displacement induced at one point of the subsoil by a load acting in another point, the elastic Mindlin solution is generally used [21].

The most important works and parametric studies conducted using BEM approaches have been carried out by Spillers and Stoll [22], Poulos and Davis [23], Davies and Banerjee [24], Sharnouby and Novak [25]; and Budhu and Davies [26,27].

The computer codes that make use of this approach include DEFPIG [28], PIGLET [29], and PGROUPN [30]. Recently some other approaches, which permit analysis of pile groups and piled-rafts [31,32,33], have been proposed.

Another interesting method is the Strain Wedge Model proposed by Ashour et al. [34,35] for the analysis of single piles and pile-groups. This method links the response of the one-dimensional beam on an elastic foundation with a three-dimensional representation of the pile-soil interaction, and thus with the development and mobilization of a passive wedge of soil in front of the pile.

## 2. Proposed ‘BEM-Based’ Method

### 2.1. Key Features of the Proposed Method

The analysis of pile-groups requires non-linear methods that have the capability to reproduce all relevant interactions between the foundation elements and the soil.

The originality of the proposed approach lies in its ability to provide a complete BEM analysis of the soil continuum in which all the interactions are modeled. Compared to FEM or FDM analyses, BEM provides a complete problem solution in terms of boundary values only, specifically at the pile-soil interfaces.

This leads to a drastic reduction in unknowns to be solved for, thereby resulting in substantial savings in computing time and data preparation effort. This feature is particularly significant for three-dimensional problems such as pile-groups.

The non-linear soil response is modeled by adopting a modified version of the quasi-hyperbolic elastic modulus reduction curve formulation proposed by Fahey and Carter [36]. The proposed method, relies upon the following assumptions:

a) pile-soil, pile-pile interactions are considered using the Mindlin’s solution;
b) horizontally layered elastic soil;
c) non-linear behavior for the reinforced concrete pile section;
d) non-linear soil behavior (incremental analysis);
e) the so-called shadowing effect, has been implemented in the code using an approach similar to that described in [35];
f) the ultimate soil pressure profile is evaluated according to the relationship suggested by [37,38,39,40].

To validate the proposed method with its main assumptions, some well-documented case histories have been collected and a prediction exercise has been carried out. The number of piles in the group studied is generally rather small (the largest pile-group studied was composed by 15 piles). In all the examined cases, a load test on a single pile was also available.

### 2.2. Pile Modelling

The proposed method was developed to capture the response of a pile group subjected to horizontal load. It consists of a BEM-based approach. The analysis is performed using a non-linear incremental tangent method.

The pile is modeled as a vertical strip, geometrically defined by the outer diameter *D* and length *L* of the actual pile, discretized in 60 blocks of variable length with depth. With this discretization, it is possible to minimize the calculation-time.

**Figure 1:**
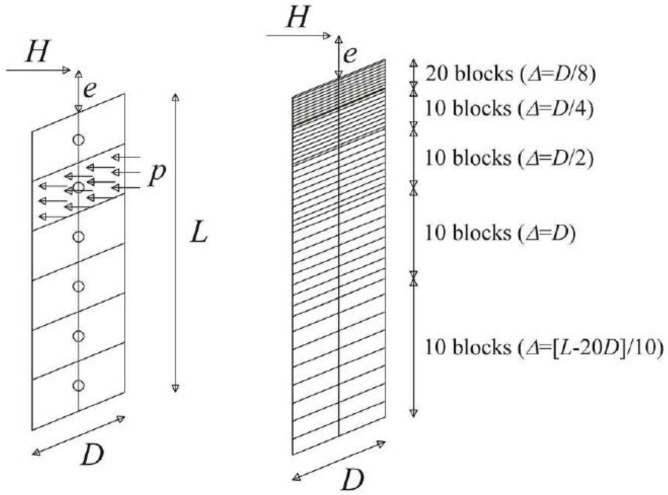
Pile discretization.

The discretization is as follows (Figure 1):

- 20 blocks with a thickness Δ = *D*/8, starting from the ground level up to a depth of 2.5*D*;
- 10 blocks with a thickness Δ = *D*/4, starting from a depth of 2.5*D* up to a depth of 5*D*;
- 10 blocks with a thickness Δ = *D*/2, starting from a depth of 5*D* up to a depth of 10*D*;
- 10 blocks with a thickness Δ = *D*, starting from a depth of 10*D* up to a depth of 20*D*;
- 10 blocks with a thickness Δ = (*L* − 20*D*)/10, starting from a depth of 20*D* up to the pile base depth.

The definition of the discretization criterion was suggested by Landi [41] as result of some parametric analyses. The same pile modeling described herein has been recently used by the authors in [42] to study the lateral response of the single pile. To facilitate the reader‘s overall understanding of the manuscript, the authors reported here some details of the pile modeling that can be also retrieved from [42].

**Figure 2:**
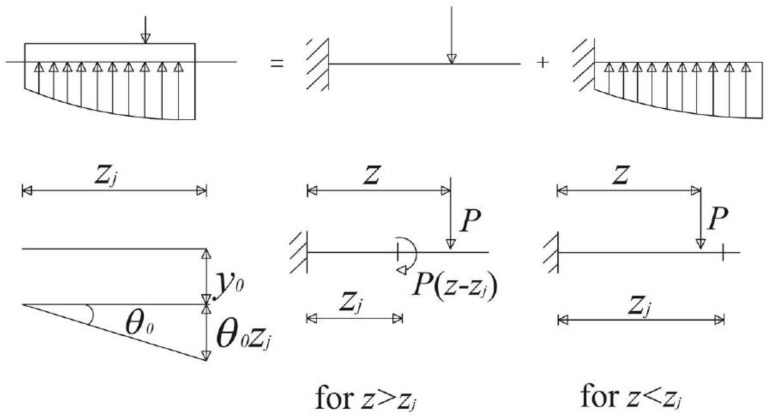
Pile flexibility matrix using the elastic beam theory (auxiliary restraint approach).

The pile flexibility matrix, in case of linear elastic behavior, is obtained using the elastic beam theory, and each coefficient of this matrix can be expressed using Equation (1) (Figure 2).

(1) $$a_{ij}=\frac{z_{i}^{3}}{3E_{p}I_{p}}+\frac{z_{i}^{2}\left(z_{j}-z_{i}\right)}{2E_{p}I_{p}}\;\mathrm{if}\;z_{i}<z_{j}\;a_{ij}=\frac{z_{j}^{3}}{3E_{p}I_{p}}+\frac{z_{j}^{2}\left(z_{i}-z_{j}\right)}{2E_{p}I_{p}}\;\mathrm{if}\;z_{i}\geq z_{j}$$

In this way, the horizontal displacement of each pile-block assumes the expression as in Equation (2).

(2) $$y_{i}=-\sum_{j=1}^{n}a_{ij}P_{j}+y_{0}+\theta_{0}z_{i}$$

in which *P_j_* represents the load applied at the generic pile-block *j* (located at depth *z_j_*), and *y*_0_ and *θ*_0_ are the unknown displacement and rotation at the pile-head. Obviously if the pile-head is fixed, the rotation becomes a known term. Each pile-point displacement is a function of *n* + 2 (or *n* + 1, for fixed condition) unknowns, *n* pile-soil interface pressures, *y*_0_ and *θ*_0_.

The proposed method analyses both steel-pipe and reinforced concrete piles. For the analysis of steel piles, the flexural rigidity *E_p_I_p_* is assumed to be constant (which means hypothesizing a linear-elastic behavior of the section until the ultimate bending moment occurs). For reinforced concrete sections, the development of cracks, even at low values of the bending moment, requires a different modeling for the pile response. In this case, the “moment-curvature-axial load” relationship is obtained by a model that has the additional feature of taking the influence of tension stiffening into account [43].

Details on this model are presented in [43], however this model represents an extension to the circular section of another model that considers the tension stiffening influence for rectangular reinforced concrete sections [44].

Once the moment-curvature relationship has been obtained, the coefficients of the flexibility matrix need to be defined using Equation (3) for the reinforced concrete pile, which is modeled in this case as a step-tapered beam with a variable flexural rigidity, *E_p_I_p_*, along the pile shaft. In Equation (3), the variation of both *E_p_* and *I_p_* along the shaft is fully considered by changing *I_p_* of the section, while *E_p_* is kept constant. Consequently, in an incremental analysis, the pile flexibility matrix needs to be updated at each load increment.

(3) $$a_{ij}=\sum_{k=1}^{i-1}\left[\left(\frac{{\left(l_{k}-l_{k-1}\right)}^{3}}{3E_{p}I_{k}}+\frac{\left(z_{j}-l_{k}\right)\cdot {\left(l_{k}-l_{k-1}\right)}^{2}}{2E_{p}I_{k}}\right)+\left(\frac{{\left(l_{k}-l_{k-1}\right)}^{2}}{2E_{p}I_{k}}+\frac{\left(z_{j}-l_{k}\right)\cdot \left(l_{k}-l_{k-1}\right)}{E_{p}I_{k}}\right)\cdot \left(z_{i}-l_{k}\right)\right]\;+(\frac{{\left(z_{i}-l_{i-1}\right)}^{3}}{3E_{p}I_{i}}+\frac{\left(z_{j}-z_{i}\right)\cdot {\left(z_{i}-l_{i-1}\right)}^{2}}{2E_{p}I_{i}})\;\mathrm{if}\;z_{i}<z_{j}\;a_{ij}=\sum_{k=1}^{j-1}\left[\left(\frac{{\left(l_{k}-l_{k-1}\right)}^{3}}{3E_{p}I_{k}}+\frac{\left(z_{j}-l_{k}\right)\cdot {\left(l_{k}-l_{k-1}\right)}^{2}}{2E_{p}I_{k}}\right)+\left(\frac{{\left(l_{k}-l_{k-1}\right)}^{2}}{2E_{p}I_{k}}+\frac{\left(z_{j}-l_{k}\right)\cdot \left(l_{k}-l_{k-1}\right)}{E_{p}I_{k}}\right)\cdot \left(z_{i}-l_{k}\right)\right]\;+\left(\frac{{\left(z_{j}-l_{j-1}\right)}^{3}}{3E_{p}I_{j}}+\frac{\left(z_{i}-z_{j}\right)\cdot {\left(z_{j}-l_{j-1}\right)}^{2}}{2E_{p}I_{j}}\right)\;\mathrm{if}\;z_{i}\geq z_{j}$$

In Equation (3), *z_i_* and *z_j_* represent respectively the distance between the fixed node in Figure 2 and the point along the beam in which the displacement is considered and the distance between the same fixed node and the point where the load is applied. On the other hand, *l_k_*, represents the distance between the fixed node and the lower part of block *k*, and *E_p_I_k_* is the flexural rigidity of block *k*.

### 2.3. Soil Modelling

The soil is modeled as a multi-layered elastic half-space. BEM analysis requires an appropriate elementary singular solution to be integrated on the surface of the problem domain. In the case of piles subjected to horizontal loading, the elastic Mindlin solution is generally used [21]. This solution, which evaluates the pile-soil interactions, is valid and rigorous only in the case of a homogeneous elastic half-space, however it can still be considered valid in the case of a multi-layered elastic half-space [23].

In this work, the approximation suggested by Poulos and Davis [23] is used, so the soil elastic modulus introduced in the Mindlin equation is the mean value between the elastic modulus at the point where the displacement is evaluated and the elastic modulus at the point where the force is applied: *E* = *(E_i_ + E_j_)*/2.

**Figure 3:**
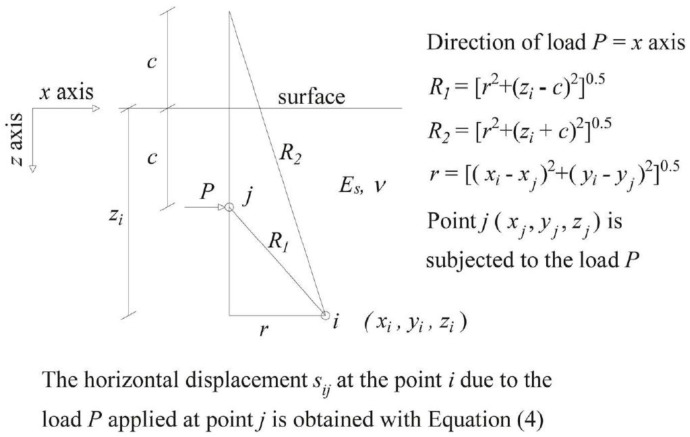
Mindlin solution scheme.

The horizontal displacement *s_ij_* at a point *i* belonging to the half space by a horizontal load *P_j_* applied at point *j* can be expressed as in Equation (4) (Figure 3). Where the term *b_ij_* represents the general expression for each soil flexibility matrix coefficient.

(4) $$s_{ij}=\frac{P_{j}\left(1+\nu \right)}{8\pi E_{s}(1-\nu )}\left[\frac{\left(3-4\nu \right)}{R_{1}}+\frac{1}{R_{2}}+\frac{x^{2}}{R_{1}^{3}}+\frac{3-4\nu }{R_{2}^{3}}x^{2}+\frac{2cz}{R_{2}^{3}}\left(1-\frac{3x^{2}}{R_{2}^{2}}\right)+\frac{4\left(1-\nu \right)\cdot \left(1-2\nu \right)}{R_{2}+z+c}\left(1-\frac{x^{2}}{R_{2}\left(R_{2}+z+c\right)}\right)\right]=b_{ij}P_{j}$$

#### Soil Non-Linear Behavior

In [45,46,47] the shear stress-strain curves were approximated with hyperbolae, with the tangent equal to *G*_max_ at zero strain and where the tangent is asymptotic to *τ*_max_ at infinite strain.

By defining a reference strain (*γ_ref_* = *τ*_max_*/G*_max_) it was possible to rewrite the equation of a hyperbola as a normalized secant shear modulus (*G*_sec_*/G*_max_) that is reduced with a normalized shear strain (*γ*/*γ_ref_*), Equation (5).

(5) $$\frac{G_{\sec}}{G_{\max}}=\frac{1}{\left(1+\frac{\gamma }{\gamma_{ref}}\right)}$$

Fahey and Carter [36], instead, used the formulations in Equation (6) and Equation (7) for the secant and the tangent elastic modulus reduction respectively.

(6) $$\frac{G_{\sec}}{G_{\max}}=1-R_{f}{\left(\frac{\tau }{\tau_{\max}}\right)}^{g}$$

(7) GtanGmax=(GsecGmax)2[1−Rf(1−g)⋅(ττmax)g]

These represent a quasi-hyperbolic relation written in terms of shear stress rather than shear strain, and employing the coefficient *g* to adjust the curve shape.

To model the non-linear behavior of the soil, therefore, a modified version of the formulation proposed by Fahey and Carter [36] was adopted.

The vertical stresses (at the pile-soil interface points) are assumed not to vary during the horizontal load analysis, so only the horizontal stresses change. An analogy can thus be assumed between the “interface pressure-ultimate soil resistance” ratio and the “shear stress–maximum shear stress” ratio ($p/p_{ult}\approx \tau /\tau_{\max}$).

With this assumption, at each step of the analysis the value of the tangent elastic modulus is updated at each pile-soil interface point using Equation (8).

(8) GtanGmax=(GsecGmax)2[1−Rf(1−g)⋅(ppult)g]

In the proposed method *R_f_* is equal to 1, while the parameter *g* ranges between 0.25 and 1. The appropriate value for *g*, to perform the analysis, can be easily estimated by trying to obtain the best fit with the load-deflection curve of a lateral load test on a single pile or with the load-deflection curve obtained with other available codes [12,14,28,30,34,42].

The input data required to define the soil flexibility matrix are: the Young’s modulus at small strain level, *E_max_* and the Poisson ratio. While the input data to define the soil resistance are: the angle of friction or the undrained shear strength for cohesionless or cohesive, respectively.

The solving scheme, is typical of BEM methods, and requires the imposition of: a) compatibility equations between the soil and pile displacements and b) equilibrium equations to translation and rotation (using appropriate boundary conditions defined at the pile-heads).

In the following, the solution system is fully described for the free-head or fixed-head pile group cases, however a different restraint condition can be considered.

The analyses are performed incrementally, using an adaptive step-size control.

### 2.4. Influence of Suction on Pile Group Response to Horizontal Loading

Suction is an important aspect in pile foundation subjected to lateral loads because the response of this foundation system is mainly affected by the shallower soil layers. The proposed method uses the “MK-Model” (Modified-Kovacs Model) proposed by Aubertin et al. [48]. This model makes use of a parameter defined as the equivalent capillary rise *h_c_*_0_ in the porous medium. The role of this parameter is the same as the average capillary rise in the original model developed by Kovacs and is calculated using the expression for the rise of water in a capillary tube (*h_c_*) with a diameter *d*.

For the sake of convenience, the expression to estimate the equivalent capillary rise in granular soils Equation (9) and in cohesive/plastic soils is reported below Equation (10).

(9) $$h_{c0}(cm)=\frac{0.75}{e\cdot D_{10}\left[1+1.17\cdot \log C_{U}\right]}$$

where *D*_10_ (in cm) is the diameter corresponding to 10% passing on the grain-size distribution curve, C_U_ is the coefficient of uniformity (=*D_60_*/*D_10_*), and *e* is the void ratio.

(10) $$h_{c0}=\frac{0.15\cdot \rho_{s}}{e}w_{L}^{1.45}$$

where *w_L_* is the liquid limit, and *ρ_s_* is the solid grain density (kg/m^3^).

The MK-Model uses the equivalent capillary rise as a reference parameter to define the relationship between the degree of saturation *S_r_* (or volumetric water content *θ*) and the matric suction ψ. The model considers that water is held by capillary forces responsible for capillary saturation *S_c_*, and by adhesive forces, causing saturation by adhesion *S_a_*. The *S_c_* component is more important at relatively low suction values, while the *S_a_* component becomes dominant at a higher suction when most capillary water has been withdrawn. The relationship proposed in the MK-Model is written as in Equation (11) for the degree of saturation:

(11) $$S_{r}=\frac{\theta }{n}=S_{c}+S_{a}^{*}\cdot \left(1-S_{c}\right)$$

In this equation, to ensure that this component does not exceed unity at low suction a truncated value of the adhesion component S_a_* is introduced in place of *S_a_* used in the original model. The contribution of the capillary and the adhesion components to the total degree of saturation is defined as a function of *h_c_*_0_ and ψ using the equations reported in Aubertin et al. [48].

Implementing the “MK-Model” in the BEM-based method takes suction into account and increases the effective stress state of the upper soil layers. This thus increases both the stiffness and the resistance of the soil located close to the ground surface, which is expressed as a function of the soil stress state.

### 2.5. Group Effects Modelling

The experimental data revealed that for small spacing values the interaction between piles belonging to different rows cannot be studied only considering a non-linear reduction of the soil elastic modulus. This is because the movements of the front piles instantaneously cause an active state condition in the soil behind the shaft. This causes not only a reduction in the stiffness of the soil responsible for the back piles response, but also a reduction in resistance.

Therefore, the proposed BEM method required of an approach to better capture the behavior seen in experimental data. The approach chosen is similar to that proposed by Ashour et al. [35].

**Figure 4:**
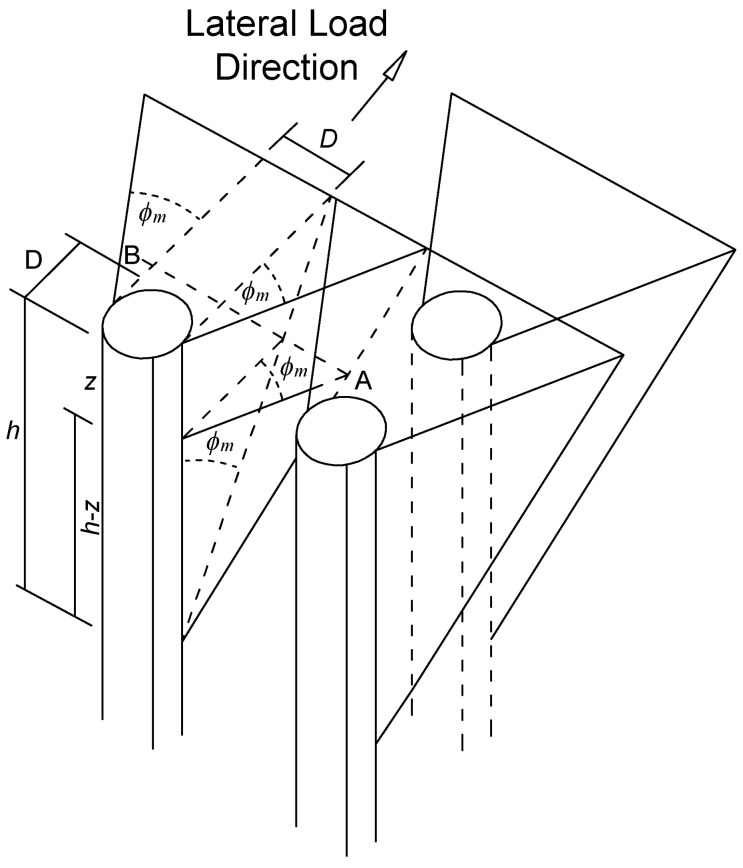
Mobilized soil passive wedges and pile-group interaction scheme (similarly as described in the context of the so-called Strain Wedge Model [35]).

In the latter work [35], the interaction among the piles in a group is determined based on the envisioned geometry of the developing passive wedge of soil in front of the pile in addition to the pile spacing. As shown in Figure 4, the soil passive wedge in front of a specific pile in the group overlaps with those of adjacent piles by an area that changes with depth.

**Figure 5:**
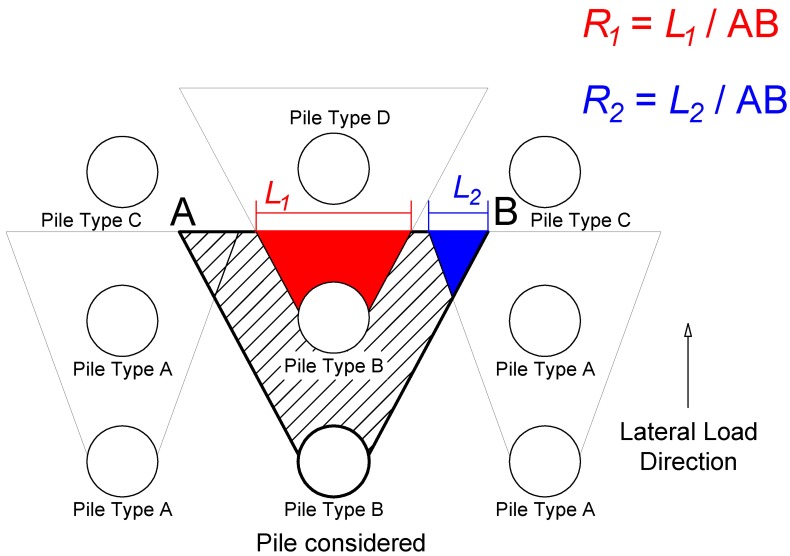
Lateral interaction for a specific pile in the group (similarly as described in the context of the so-called Strain Wedge Model [35]).

The overlap of these wedges of neighboring piles at a generic depth *z* is characterized as shown in Figure 5.

According to the pile classification shown in Figure 5, the load carried by inner piles is less than the load carried by the outer piles in each row. This fact was observed in several field tests as described in [35].

As stated in [35], at a given depth (Figure 5), overlapping areas exhibit larger values of soil strains and stresses compared to the isolated pile. The increase in the average soil stress attributable to the passive wedge of a given pile depends on the number and area of interfering wedges overlapping the wedge of the pile in question (Figure 5).

This overlap depends on the position of the pile in the group.

The average stress level in a soil layer (*SL_g_*) due to passive wedge interference is evaluated based on an empirical relationship Equation (12), which provides good agreement with field test results [35].

(12) $$SL_{g}=SL_{i}{\left(1+\sum R_{j}\right)}^{1.5}\leq 1$$

where *j* = number of neighboring passive wedges in soil layer *i* that overlap the wedge of the pile in question; *R* = ratio between the length of the overlapped portion of the face of the passive wedge (*L*) and the total length of the face of the passive wedge (AB); and *R_j_* is determined from all the neighboring piles (sides and front piles) of the pile in question (Figure 5).

The *SL_i_* value on the right-hand side of Equation (12), which represents the *SL* of the single isolated pile, for cohesionless soils in the Strain Wedge model, is defined in Equation (13).

(13) $$SL=\frac{\Delta \sigma_{h}}{\Delta \sigma_{hf}}=\frac{\tan^{2}\left(45^{{}^{\circ}}+\varphi_{m}/2\right)-1}{\tan^{2}\left(45^{{}^{\circ}}+\varphi /2\right)-1}$$

where the horizontal stress change at failure Δ*σ*_h*f*_ (or the deviatoric stress at failure in the triaxial test) is $\Delta \sigma_{hf}=\sigma_{v0}\left[\tan^{2}\left(45^{{}^{\circ}}+\varphi /2\right)-1\right]$. However, in the proposed method it is assumed that $SL_{i}≅p/p_{ult}$, and thus:

(14) $$\frac{p}{p_{ult}}≅\frac{\tan^{2}\left(45^{{}^{\circ}}+\varphi_{m}/2\right)-1}{\tan^{2}\left(45^{{}^{\circ}}+\varphi /2\right)-1}$$

The mobilized friction angle, *ϕ_m_*, can be easily obtained if *SL_i_* is known, which is assumed to be approximately equal to the ratio $p/p_{ult}$.

The values of *SL_g_* vary with depth and level of loading. They can be used to evaluate the increased value of the pressure at each pile-soil interface ($p_{g}$) (where this increase is caused by the interferences of the passive wedges) Equations (15) and (16).

(15) $$SL_{g}=\frac{p}{p_{ult}}{\left(1+\sum R_{j}\right)}^{1.5}$$

(16) $$SL_{g}=\frac{p_{g}}{p_{ult}}$$

The value assumed by $p_{g}$ at each pile-soil interface is then used to update the value of the tangent elastic modulus of the soil at each depth using Equation (8) in which *p* = *p_g_*.

**Figure 6:**
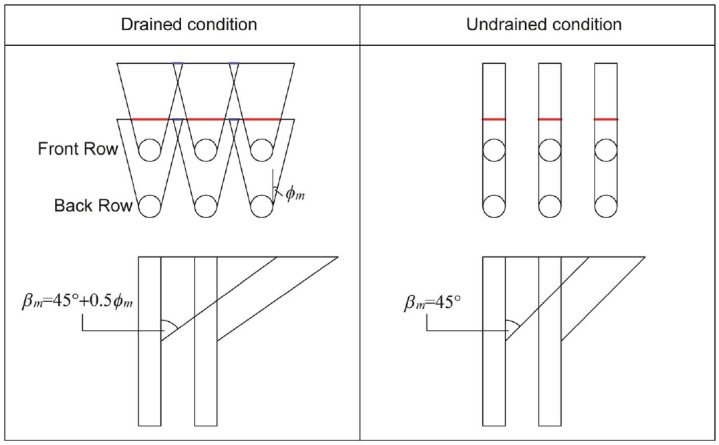
Pile group effect: shadowing modeling.

For cohesive soils, on the other hand, is assumed to be in an undrained-condition (total stress). Consequently, the value of *ϕ* is equal to 0° and also the value of ϕ_m_ is always 0°. This means that the base angle of the passive wedge, for cohesive soils, is constantly equal to 45° and only the dimension in depth (and thus on the plain) of the passive wedge changes when the load increases. However, in this way only the interaction between the wedge of a pile positioned in a row different from the front row with the wedge of the pile located in front of it can be considered, and thus the interactions between the wedges of piles belonging to the same row are neglected (Figure 6).

To overcome this limit, and thus to consider the interactions between the wedges of piles located side by side, we consider the extreme case in which a row of piles has a relative spacing *s/D* equal to 1. In this condition, theoretically, the ultimate soil resistance profile should be coincident to the one in a retaining wall, given by the difference of the passive earth pressure and the active earth pressure in an undrained condition. In this case, the active and passive soil pressure profiles (in terms of force per unit length) acting along the pile shaft are expressed by Equations (17) and (18) respectively.

(17) $$p_{a}\left(z\right)=\left[\sigma_{v0}\left(z\right)-2c_{u}\left(z\right)\right]\cdot D=\left[\gamma \cdot z-2c_{u}\left(z\right)\right]\cdot D$$

(18) $$p_{p}\left(z\right)=\left[\sigma_{v0}\left(z\right)+2c_{u}\left(z\right)\right]\cdot D=\left[\gamma \cdot z+2c_{u}\left(z\right)\right]\cdot D$$

**Figure 7:**
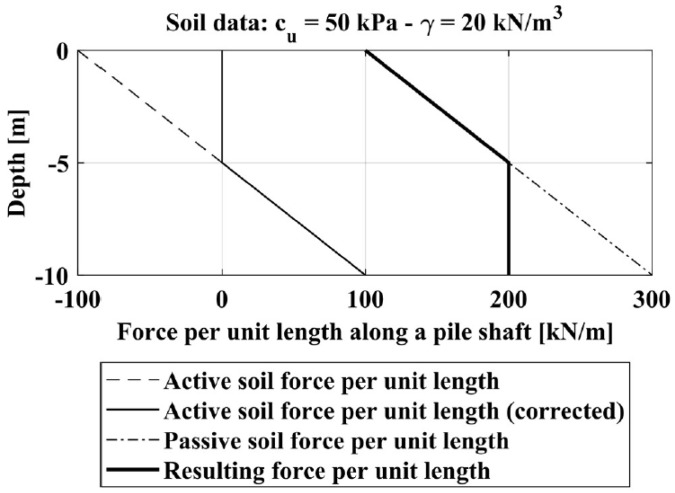
Soil pressure profile.

Note that the value of *p_a_*, for shallow depths, could be negative and this means that the soil is in tension. Assuming reasonably that the soil cannot support tension all the values of *p_a_* < 0 are corrected considering directly *p_a_* = 0. The difference between *p_p_* and *p_a_* thus represents the ultimate soil pressure profile, *p_r_*, (in terms of force per unit length) acting along a pile shaft in a row of piles with a spacing of 1*D* (or on a retaining wall). For example, Figure 7 shows all these steps to define the resulting lateral pressure profile, *p_r_*, considering a homogenous cohesive soil with a constant *c_u_* equal to 50 kPa, a soil unit weight γ equal to 20 kN/m^3^ and a pile diameter *D* = 1 m.

The soil resistance profile is now considered, for the same soil condition shown before, as defined by Matlock [37] for a single isolated pile in soft clay, and thus expressed by the minimum values of Equation (19).

(19) $$\min\left\{\left[3+\frac{\gamma \prime }{c_{u}}z+\frac{J}{D}z\right]c_{u};9c_{u}D\right\}$$

**Figure 8:**
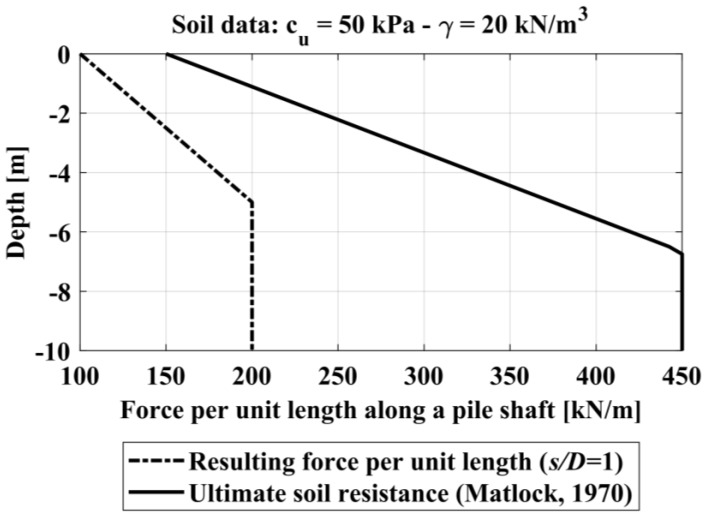
Resulting soil pressure profile.

Figure 8 compares the values of *p_r_* (for a spacing of 1*D*) and *p_ult_* (for a single isolated pile).

Based on all the experimental data studied, it is assumed that the ultimate soil resistance profile for the single isolated is valid only for pile spacing *s/D* ≥ 6. For a pile spacing ratio of less than 6 it is considered that the ultimate soil resistance profile is intermediate to the profile *p_r_* (assumed for spacing ratio s*/D* = 1) and the profile *p_ult_* (assumed for spacing ratio *s/D* ≥ 6).

To evaluate the definitive soil resistance profile (*p_ult,def_*), for spacing ratio between 1 and 6, it is assumed that *p_ult,def_* can be expressed as a function of the actual spacing ratio s/D and the depth, z, using this relationship in Equation (20).

(20) $$p_{ult,def}\left(z\right)=p_{r}\left(z\right)+F\left(s/D\right)\cdot \left(p_{ult}\left(z\right)-p_{r}\left(z\right)\right)$$

where, *F(s/D)* is a factor defined as a function of the relative spacing ratio according to Equation (21).

(21) $$F\left(s/D\right)=\frac{s/D-1}{5}$$

With this procedure, even for cohesive soil in undrained condition, it is possible to consider the interactions between piles located side by side, simply by substituting the ultimate soil resistance evaluated with the expressions defined by Matlock [37] with the *p_ult,def_* values.

In the proposed approach, the mobilized soil passive wedge starts to develop at a depth, along the pile shaft, which is different from pile to pile, where the pressure at the interface changes from a positive to a negative value, passing from a passive to an active state.

### 2.6. Solution System

The solution system is defined as: [*F*][*X*] = [*P*] Equation (22). [*X*] is the unknowns vector made up of *km* + 2*m* + 1 terms or *km + m* + 1 terms for free or fixed head conditions, respectively; where *m* is the number of piles, *k* the number of the pile blocks for each pile, *p* are the *km* unknown pressures acting at the pile-soil interfaces, *y_0_* is the pile-group displacement, *θ_m_* are the *m* pile-heads rotations, *H_m_* are the *m* horizontal loads at the pile-heads and [*P*] is the known-terms vector (with the same dimension as for the vector [*X*]). [*F*] is a (*km* + 2*m* + 1) *×* (*km* + 2*m* + 1) or (*km + m* + 1) *×* (*km + m* + 1) matrix, obtained by summing:

- the *km × km* pile flexibility matrix [*F_P_*], composed of the *a_ij_* coefficients;
- the *km × km* flexibility matrix [*F_S_*], composed of the *b_ij_* coefficients that represent the displacements induced by a load acting at the pile-soil interface *j* to the pile-soil interface *i*.

The last 2*m* + 1 or *m* + 1 rows and columns, of the [*F*] matrix, are necessary to impose the equilibrium and to complete the compatibility equations at each pile-soil interface node. In Equation (22), *H* is the applied horizontal load and *f* is the load eccentricity.

The flexibility matrix [*F*] is updated at each step of the procedure. The pile flexibility sub-matrix, [*F_P_*], is updated only in the case of a non-linear “moment-curvature” relationship for the pile section. [*F_P_*] is updated using the tangent flexural rigidities, according to the bending-moments reached at each pile-node in the previous load increment.

(22) $$\left[\begin{array}{cccccccccc} a_{1,1}+b_{1,1} & \cdots & a_{1,km}+b_{1,km} & -1 & -z_{1,1} & \cdots & \cdots & 0 & \cdots & 0\\ \vdots & \ddots & \vdots & \vdots & \vdots & \ddots & \vdots & \vdots & \ddots & \vdots \\ a_{km,1}+b_{km,11} & \cdots & a_{km,km}+b_{km,km} & -1 & \cdots & \cdots & -z_{km,m} & 0 & \cdots & 0\\ 1 & \cdots & 1 & 0 & 0 & \cdots & 0 & 0 & \cdots & 0\\ z_{1,1} & \cdots & \cdots & 0 & 0 & \cdots & 0 & 0 & f & f\\ \vdots & \ddots & \vdots & \vdots & \vdots & \ddots & \vdots & f & 0 & f\\ \cdots & \cdots & z_{m,km} & 0 & 0 & \cdots & 0 & f & f & 0\\ 1_{1,1} & \cdots & \cdots & 0 & 0 & \cdots & 0 & -1 & 0 & 0\\ \vdots & \ddots & \vdots & \vdots & \vdots & \ddots & \vdots & 0 & -1 & 0\\ \cdots & \cdots & 1_{m,km} & 0 & 0 & \cdots & 0 & 0 & 0 & -1 \end{array}\right]\left[\begin{array}{c} {p_{1}}^{p}\cdot \Delta_{1}D\\ \vdots \\ {p_{km}}^{p}\cdot \Delta_{km}D\\ y_{0}\\ \theta_{1}\\ \vdots \\ \theta_{m}\\ H_{1}\\ \vdots \\ H_{m} \end{array}\right]=\left[\begin{array}{c} 0\\ \vdots \\ 0\\ H\\ fH\\ \vdots \\ fH\\ 0\\ \vdots \\ 0 \end{array}\right]$$

**Figure 9:**
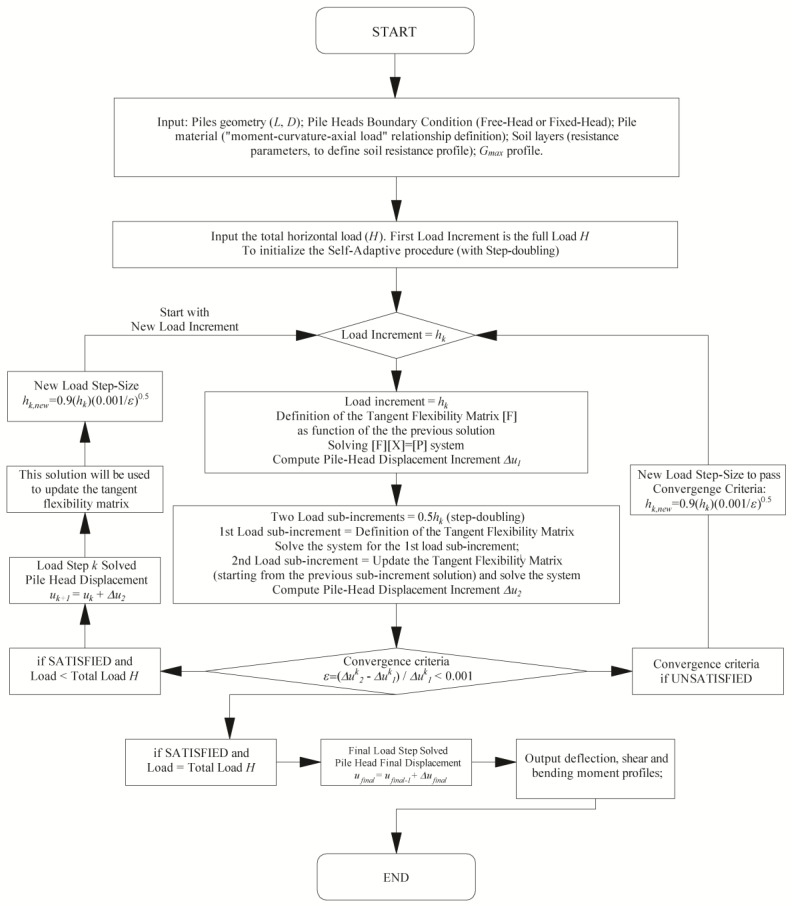
Flow chart of the proposed non-linear adaptive step-size method.

Once the initial flexibility matrix, [*F*], has been computed, the total horizontal load is applied in the first step of the solution procedure. At each generic load increment *h_k_*, an iterative process is performed where two solutions are obtained, the first using *h_k_* as the load increment, the second using two load steps equal to *h_k_*/2. The iterative scheme is described in Figure 9, which, for the sake of simplicity refers to the explicit Euler method with step-doubling and adaptive step-size control. However, a fourth order Runge-Kutta method can also be used to obtain some improvement in the accuracy of the solution. The adaptive step-size control numerical technique is fully described in Press et al. [49].

**Figure 10:**
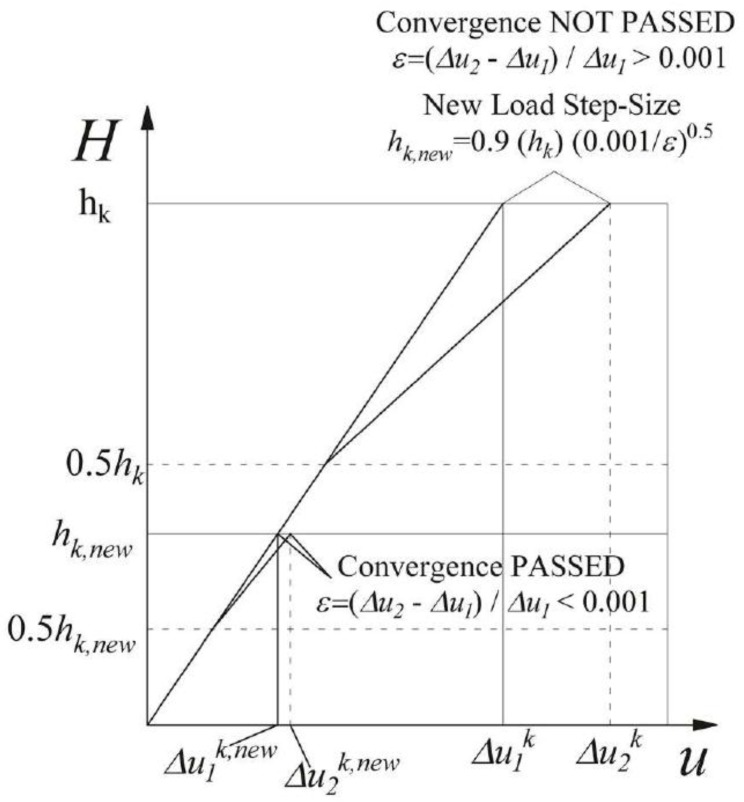
Adaptive step-size control.

Once these two solutions have been computed, the incremental ratio *ε*, is computed according to Equation (23).

(23) $$\varepsilon =\frac{\Delta u_{2}-\Delta u_{1}}{\Delta u_{1}}$$

where *Δu*_1_ and *Δu*_2_ are the incremental displacement at the pile-head evaluated using one and two steps, respectively. The *ε* value is compared with a predefined tolerance taken as equal to 0.001 (Figure 10).

When this convergence criterion is exceeded (*ε* > *tol*), the iterative process starts again with an updated load increment *h_k_^new^* which should be able to achieve the desired accuracy and can be estimated using Equation (24) [49].

(24) $$h_{k}^{new}=SF\cdot h_{k}\cdot {\left(\frac{tol}{\varepsilon }\right)}^{\frac{1}{p+1}}$$

where *p* is the order of the method used (in the Euler method *p =* 1, in the Runge-Kutta method *p =* 4), and *SF* is a “safety factor” (taken as equal to 0.90) to guarantee the success in the next attempt. When this convergence criterion is passed (*ε* ≤ *tol*), Equation (24) is used again to estimate the next step-size. The procedure stops when the final lateral load *H* is reached. Finally, the entire load-deflection curve and the deflection, shear, bending moment and pile-soil interface pressure profiles along the pile shaft at each load-step can be evaluated.

## 3. Validation of the Proposed Method

In this section, are presented the results of the pile group analyses using the BEM-based approach proposed within this paper. These results are compared with those obtained in horizontal load tests on pile groups in sandy and cohesive/plastic soils. The lateral load tests were carried out both on steel and r.c. piles. The experimental data were retrieved from well-documented tests in the available literature for a total of 15 pile groups case histories, reported in Table 1. Further information about the validation procedure can be found in [50].

In the case histories studied the maximum number of piles in the group is 15 and the lateral load test data on a single pile were always included.

The purpose of the analyses is to validate the BEM approach developed in this work. The analyses were realized as class A predictions by using the actual pile properties and the soil geotechnical parameters obtained based on the in situ and laboratory tests data. In particular, the soil elastic modulus to be considered is a Young’s Modulus at small strain level inferred from in situ tests*.*

It needs to be remembered that in the analyses of pile-groups the value of the exponent *g* of the elastic modulus reduction curve must be defined. The appropriate *g* value to be inputted can be easily estimated trying to obtain the best fit with the load-deflection curve of the horizontal test on the single pile. In Table 2 are reported the input data used to perform the analyses with the proposed method. These data refer to the properties of the soil layers at least in between the ground surface and 10-diameter depth. Additional details are presented in [50].

**Figure 11:**
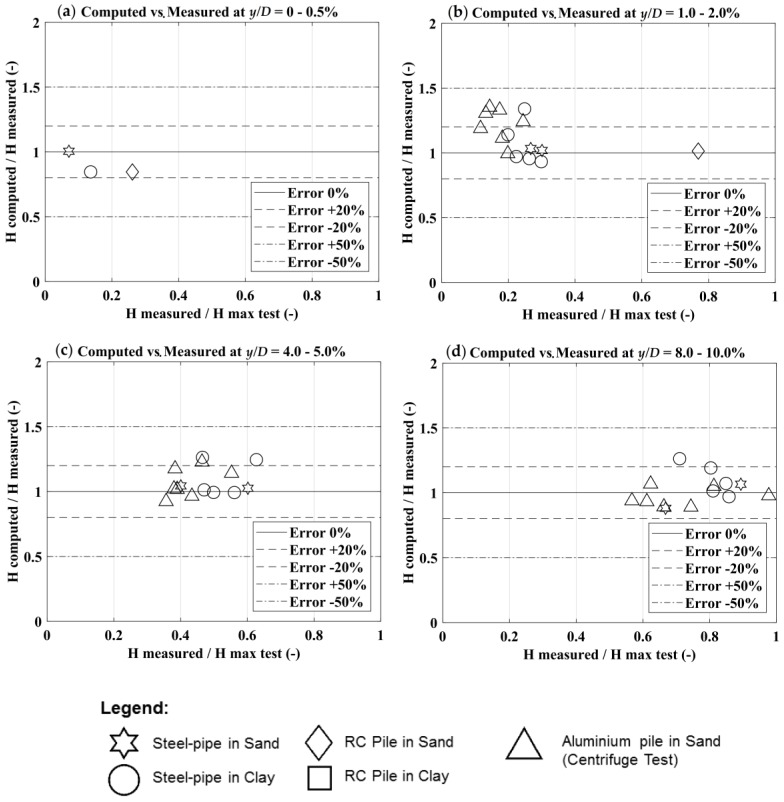
Measured vs. computed horizontal loads (H) for a given normalized displacement (*y/D*): (**a**) *y/D* = 0–0.5%; (**b**) *y/D* = 1.0–2.0%; (**c**) *y/D* = 4.0–5.0%; (**d**) *y/D* = 8.0–10.0%.

The comparison between computed and measured results (Figure 11) clearly show the capability of the proposed BEM method to provide good predictions of the pile groups lateral response.

In Figure 11, the ratio among the experimental lateral load for a given normalized displacement (*y/D*) and the experimental maximum horizontal load is on the *x-axis*, while the ratio among the calculated and the measured load (for a given *y/D*) is on the *y*-axis.

The error in the load forecasting at each *y/D* reached during the tests is included in the range of ±30%.

### 3.1. Analysis Results with the Proposed BEM Method for a Specific Lateral Load Test on a Bored Pile Group

A full-scale lateral load test program [4] was realized in Taiwan in 2001. Two pile groups, one consisting in bored piles and the other in driven piles, were subjected to horizontal loading tests. The tests were also conducted on single piles installed using the same two techniques. In this section, all the presented results refer to the free-head bored single pile and to the fixed-head bored pile group. The latter is a 3 × 2 (3 rows and 2 columns) pile group at 3*D* spacing.

#### 3.1.1. Soil and Pile Properties Description

The soil in the site was classified as silt or silty sand, with occasional layers of silty clay. The water table was at 1.0 m below the ground surface and did not change considerably during the entire test program period. In [4] are fully presented the cone penetration tests and *G*_max_ data profiles.

In the site were placed 13 cast in situ bored piles and 13 precast driven piles. Eleven of the 13 bored piles (*D* = 1500 mm, L = 34.9 m; *EI* = 6.86 GNm^2^) were realized using bentonite-mud with reverse circulation. Two of the 13 bored piles were realized by means of a drilling device with hydraulic oscillator at full length. The measurement instruments (strain gauges and inclinometers) were attached to the longitudinal reinforcement bars, inserted into the hole before casting the concrete. Bored pile properties are summarized in Table 3.

#### 3.1.2. Single Bored Pile B7 (Free-Head) and Pile Group (Fixed-Head): Analysis Results

The soil unit weight (*γ*) was evaluated based on the cone penetration tests data fully reported in [4]. Along the first 15 meters in depth the tip resistance in the CPT tests was on average equal to 5 MPa. The pile properties used to perform the BEM analyses are those reported in Table 3.

**Figure 12:**
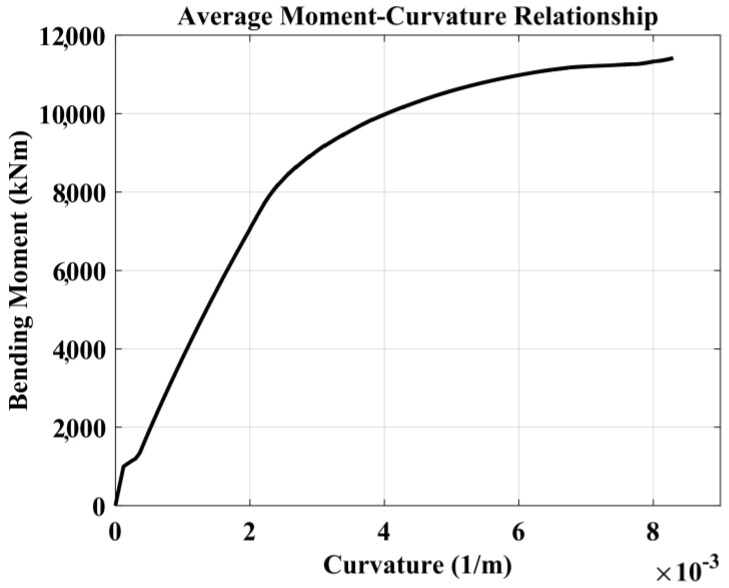
Pile section ‘bending moment-curvature’ relationship for B7 pile section.

The bending moment-curvature relationship of the pile section (Figure 12) was computed with the model that can consider the influence of tension stiffening [43].

Based on the cone penetration tests data in [4], an angle of internal friction of 34° was obtained using the correlation suggested by Mayne [55] Equation (25).

(25) $$\varphi \prime =17.6+11\cdot \log_{10}\left(\frac{q_{t}-\sigma_{v0}}{\sqrt{\sigma \prime_{v0}p_{a}}}\right)$$

The *G*_max_ profile was that provided in [4]. This profile was simplified and assumed linearly increasing from 15 to 150 MPa. The Poisson ratio was set equal to 0.35.

The ultimate soil pressure profile was evaluated according to relationship proposed by Reese et al. [38]. Since the water table was located 1 m below the ground surface, approximate suction effects were considered, because of the lack of information to use the Modified Kovacs Model rigorously, thus increasing the vertical effective soil stresses at the first meter in depth. In fact, a linearly increasing suction value was assumed from 0 kPa to 10 kPa starting from 1.0 meter depth up to the ground surface.

As a consequence, the soil resistance profile, obtained with the relationships suggested in [38], is increased in the first meter depth.

**Figure 13:**
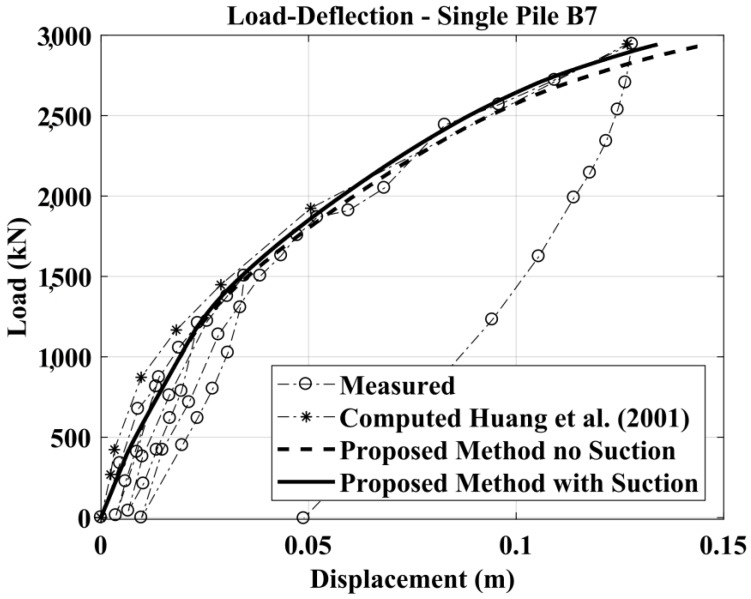
Measured vs. computed results: Lateral Load versus Head Deflection curve.

The comparison between measured and calculated results for the free-head single pile case are shown in Figure 13 (considering and not considering suction).

**Figure 14:**
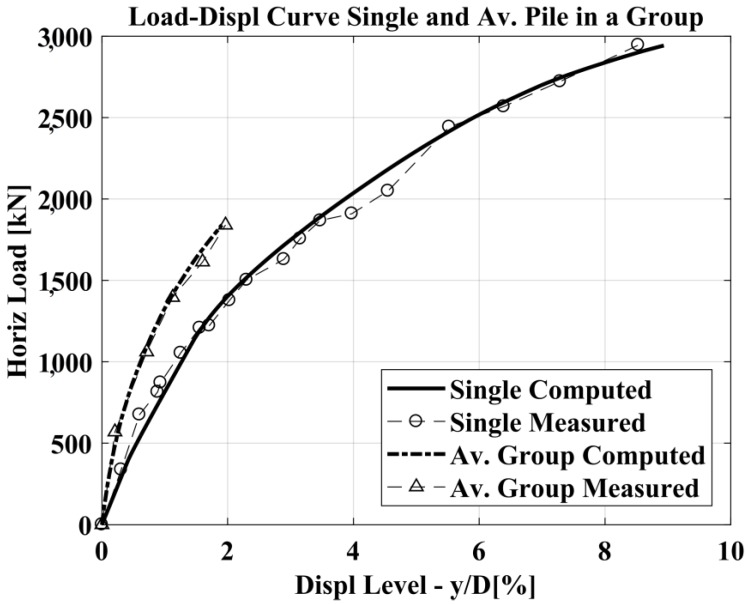
Computed vs. measured load-deflection curves.

**Figure 15:**
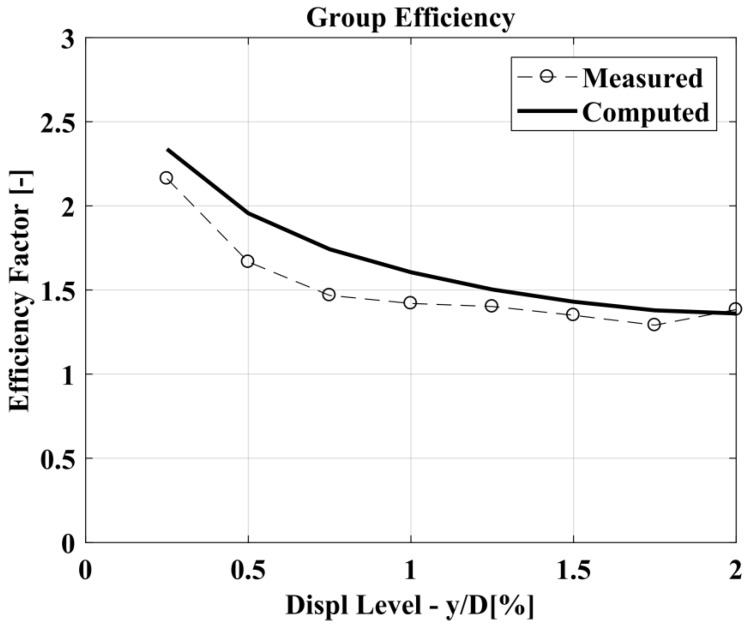
Computed vs. measured group efficiency.

**Figure 16:**
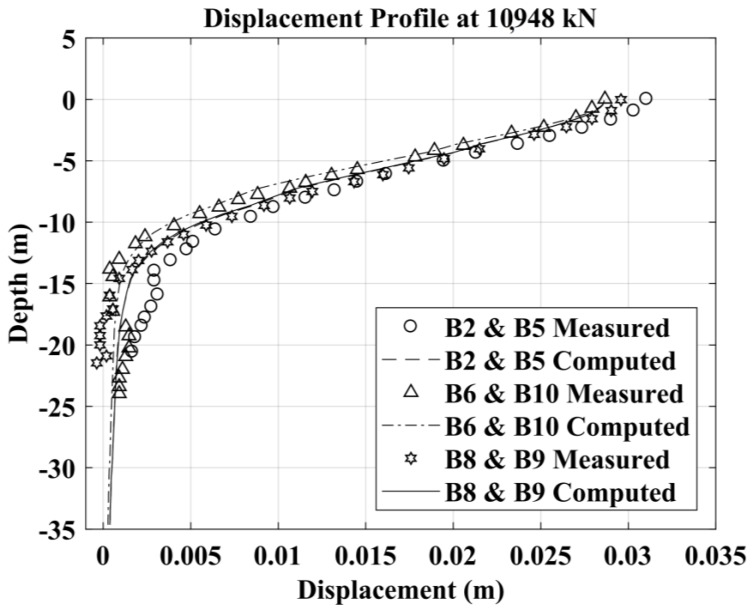
Computed vs. measured deflection profiles at *H* = 10948 kN of piles B2, B5, B6, B8, B9, B10.

The capability of the BEM method to forecast the laterally loaded pile response is good in both cases, nevertheless it can be observed an improvement in the prediction considering suction. The results for the 3 × 2 fixed-head pile group are presented in Figure 14, Figure 15 and Figure 16 in terms of the load deflection curve for an average pile in the group, group efficiency and the pile-deflection profile at a specific value of the lateral load for all the piles in the group. The group efficiency in the pile-group analysis results is defined as: *H_group_*/(*n H_single_*); where, *H_group_* = the total horizontal load in the pile group, *H_single_* = the horizontal load in the isolated single pile (at the same displacement-level) and *n* = the number of piles in the group.

The group efficiency in Figure 15 is higher than one because the single pile was tested in free-head restraint condition, while the pile group is fixed-head.

The reader should note that in [4] and in [56] the load of 1462 kN was considered as the lateral load corresponding to the first cracking bending moment based on back-analysis results.

For this reason, in [4] and more recently in [56] a reduced bending stiffness was set to the relevant section of the piles to consider cracking.

The computer codes used in [4] and [56] were LPILE [57] and VERSAT-P3D [58], respectively. The proposed BEM approach, instead, automatically updates each pile-block bending stiffness according to the “average moment-curvature” relationship obtained from the actual pile section properties.

With the proposed method, the analysis to obtain the bored pile group results requires less than 10 min to compute the entire lateral load-deflection curve on a laptop with an Intel Core i7 CPU processor (2.20 GHz). Analyses of similar problems by VERSAT-P3D [58] using a coarse finite element mesh requires about 20 min to calculate the displacement for a single point on the load-deflection curve, while FLAC-3D takes about 6 h [59].

## 4. Conclusions

A pile group subjected to horizontal load is a complex soil-structure interaction (SSI) problem affected by pile and soil non-linear behavior. Even nowadays, this specific SSI problem cannot be easily solved, especially because most of the computer codes are specialized to solve/study either structural or geotechnical issues.

One of the key aspects of the pile group lateral behavior is the continuous pile-soil relative stiffness variation, while a horizontal load is applied.

To capture the latter, a new BEM-based approach for the analysis of laterally loaded pile group has been developed and validated. Herein, the solution system of the proposed method is fully presented for the free-head and fixed-head pile group cases, however a different restraint condition can be considered.

The proposed BEM approach is innovative because can take into account for the highly non-linear behavior of reinforced concrete piles, considering also the tension stiffening effect. Moreover, the influence of suction in the upper soil layers, is also considered, by means of the Modified-Kovacs Model.

The method developed herein presents two significant merits compared to some FDM, FEM and quasi-3D FEM codes: the reduction of computation (or running) time, and the easiness in the selection/definition of the input parameters to perform the analyses.

The reliability of the proposed BEM method was tested by comparing computed and experimental data from full scale and centrifuge tests on 15 pile groups, retrieved from the available technical literature about this topic. The results presented herein have shown the capability of the BEM method to provide a good prediction both qualitatively and quantitatively of the relevant aspects of the pile group horizontal behavior. The prediction errors are lower than 30% for most of the case histories studied.

Finally, for comparison purpose, the proposed method was used to analyze a specific pile group horizontally loaded in a full-scale test program realized in Taiwan in 2001.

## Figures and Tables

**Table 1:** Case histories studied.

Case Reference	Pile Material	Pile Diameter (m)	Pile Length (m)	Soil Type	*H_max_* (kN)
[51] 3 × 3; *s* = 3*D*	Steel with Grout-fill	0.273	13.11	OC Clay	695
[5] 3 × 3; *s* = 3*D*	Steel with Grout-fill	0.273	13.11	Sand	808.5
[4] 3 × 2; *s* = 3*D*	Bored RC	1.5	34.9	Silty Sand	11043
[8] ϕ’ = 34°; 3 × 3; *s* =3*D*	Aluminum	0.43	13.3	Sand	761.2
[8] ϕ’ = 39°; 3 × 3; *s* = 3*D*	Aluminum	0.43	13.3	Sand	1508.2
[8] ϕ’ = 34°; 3 × 3; *s* = 5*D*	Aluminum	0.43	13.3	Sand	1110.5
[8] ϕ’ = 39°; 3 × 3; *s* = 5*D*	Aluminum	0.43	13.3	Sand	1424
[52] 2 × 1; *s* = 2*D*	Aluminum	0.72	12	Sand	1183
[52] 2 × 1; *s* = 4*D*	Aluminum	0.72	12	Sand	1220.1
[52] 2 × 1; *s* = 6*D*	Aluminum	0.72	12	Sand	1030.72
[53] 3 × 3; *s* = 3*D*	Steel with Grout-fill	0.305	8.7	Clay	927.05
[11] 3 × 3; *s* = 3*D*	Steel pipe	0.324	11.5	Sand	488.6
[54] 3 × 3; *s* = 5.65*D*	Steel pipe	0.324	11.9	Clay	1407
[54] 3 × 4; *s* = 4.4*D*	Steel pipe	0.324	11.9	Clay	1353.8
[54] 3 × 5; *s* = 3.3*D*	Steel pipe	0.324	11.9	Clay	1942.5

**Table 2:** Input data used to perform the analyses with the proposed method.

Case	*E_p_I_p_* (MNm^2^)	*γ* (kN/m^3^)	*ϕ* (°)	*D_R_* (%)	*c_u_* (kPa)	*E_max_* (Linear Increasing with Depth) (MPa)	*G* (-)	*F* (m)	W.T. (m)	Head B.C.
[51]	16.0	19.0	-	-	58–145 (0–5.5 m)	70–200 (0–5.5 m)	0.25	0.305	0.0	Free
[5]	16.0	19.5	47	90	-	35–100 (0–2.0 m)	1.0	0.305	0.0	Free
[4]	variable	18.5	34	50	-	40–400 (0–34.9 m)	0.5	1.0	1.0	Fixed
[8]	72.1	14.51	34	33	-	60–300 (0–13.3 m)	0.25	1.68	-	Free
[8]	72.1	15.18	39	55	-	50–260 (0–13.3 m)	0.5	1.68	-	Free
[52]	514.0	16.3	40	89	-	40–200 (0–12.0 m)	1.0	1.6	-	Free
[53]	26.91	19.0	-	-	50–75 (0–2.9 m)	60–170 (0–8.7 m)	0.25	0.4	0.0	Free
[11]	30.03	19.5	40	44	-	20–150 (0–11.5 m)	0.25	0.86	0.0	Free
[54]	30.03	19.0	-	-	60 (0–1 m)120 (1.0–4.0 m)	50–60 (0–4.0 m)	0.25	0.48	1.0	Free

Table notes: *E_p_I_p_* = pile flexural rigidity; *γ* = soil unit weight; *ϕ* = peak friction angle; *D_R_* = relative density; *c_u_* = undrained shear strength; *E_max_* = soil elastic modulus at small strain level; *g* = parameter of the modulus reduction curve; *f* = load eccentricity; W.T. = water table depth below the ground surface; Head B.C. = pile-head boundary conditions (free-head or fixed head).

**Table 3:** Structural properties of bored pile.

Pile Diameter *D* (mm)	Pile Length (m)	Cross Sectional Area (cm^2^)	Concrete Compressive Strength *f*’_c_ (MPa)	Reinforcement Yield Stress *f*_y_ (MPa)	Steel Ratio ρ_s_	Intact Flexural Rigidity *EI* (GNm^2^)
1500	34.9	17672	27.5	471	0.025	6.86
